# Serological and Molecular Investigation of Brucellosis in Breeding Equids in Pakistani Punjab

**DOI:** 10.3390/pathogens9090673

**Published:** 2020-08-19

**Authors:** Amjad Hussain, Tariq Jamil, Abdul Malik Tareen, Falk Melzer, Muhammad Hammad Hussain, Iahtasham Khan, Muhammad Saqib, Ali Zohaib, Riaz Hussain, Waqas Ahmad, Mudassar Iqbal, Heinrich Neubauer

**Affiliations:** 1 Department of Microbiology, Faculty of Life Sciences, University of Baluchistan, Quetta 87550, Pakistan; abdulmaliktareen@yahoo.com; 2 Institute of Bacterial Infections and Zoonoses, Friedrich-Loeffler-Institut, 07743 Jena, Germany; falk.melzer@fli.de (F.M.); heinrich.neubauer@fli.de (H.N.); 3 Vance Street, Bardia 2565, Australia; m.hammad.hussain@gmail.com; 4 Section of Epidemiology and Public Health, Department of Clinical Sciences, University of Veterinary and Animal Sciences, Lahore Sub-Campus Jhang 12-Km Chiniot Road, Jhang 35200, Pakistan; iahtasham.khan@uvas.edu.pk; 5 Department of Clinical Medicine and Surgery, Faculty of Veterinary Sciences, University of Agriculture, Faisalabad 38400, Pakistan; drsaqib_vet@hotmail.com; 6 Department of Healthcare Biotechnology, Atta-ur-Rehman School of Applied Biosciences, National University of Science and Technology (NUST), Islamabad 44000, Pakistan; alizohaib7@hotmail.com; 7 Department of Pathology, Faculty of Veterinary and Animal Sciences, The Islamia University of Bahawalpur, Bahawalpur 63100, Pakistan; dr.riaz.hussain@iub.edu.pk (R.H.); mudassar.iqbal@iub.edu.pk (M.I.); 8 Department of Clinical Sciences (Epidemiology), Khan Bahadar Choudhry Mushtaq Ahmed College of Veterinary and Animal Sciences, Narowal 51600, Pakistan; waqas.hussain@uvas.edu.pk

**Keywords:** *Brucella abortus*, brucellosis, equines, zoonosis, Pakistan

## Abstract

Brucellosis is an important zoonosis worldwide. Equines are susceptible to the infection when in close contact with infected animals. The objective of our study was to update the existing knowledge and detect and differentiate the causative agent of brucellosis in breeding equines in Punjab, Pakistan. A cross-sectional study was designed to evaluate the occurrence and etiology of the infection in the equine population in three districts. A total of 448 equine sera were collected from three prefectures viz. Sahiwal, Khanewal, and Okara of the Punjab Province of Pakistan. Ninety-six (21.4%) samples were found positive by RBPT, 3.56% (16/448) by iELISA, and 4.24% (19/448) by CFT. Real-time PCR demonstrated the presence of *Brucella abortus*-DNA in sero-positive samples. Age and location were found as risk factors. The study concludes equine brucellosis seroprevalence in the country where *Brucella abortus* as the main etiology. Fistulous withers and poll evil cases should be treated with care as they could be hazardous and a source of zoonotic transmission. Routine screening at an early age, vaccination in ruminants, and consumption of pasteurized dairy milk in humans is recommended for prevention of the infection. Specific tests need to be standardized and validated.

## 1. Introduction

Brucellosis is an abortive bacterial zoonosis primarily seen in ruminants and is caused by *Brucella (B.) abortus* and *B. melitensis* [1,2,3]. Transmission occurs via direct contact with infected animals or indirectly via the contaminated environment. Humans contract brucellosis mainly through consumption of unpasteurized contaminated dairy milk [4]. “Nonpreferred” hosts (e.g., equids) are susceptible when in close contact with the infected “preferred” hosts [5,6,7,8]. In domestic ruminants, abortion storms during the last trimester, hyperthermia, and retention of fetal membranes and then intermittent fever, arthralgia, and myalgia in acute cases and neurologic disorders in chronic cases in humans are characteristic signs [9]. Equines are potential dead end-host of brucellosis as infection remains asymptomatic with secondary signs (e.g., supra-atlantal bursitis, fistulous withers, carpal hygroma, olecranon bursitis) and occasional epididymitis in stallions [10,11,12,13]. The bursal fluid from such equids may be a source of infection in humans. In Pakistan, brucellosis has been reported in ruminant herds caused by both *B. abortus* and *B. melitensis* [1,5]. In humans, occupational exposure to the infected animals has also been associated with transmission of the infection in the country [14,15,16]. Equines are a source of livelihood for poor families and are also status symbols for institutions and rich people in Pakistan [17]. The main purpose of equine rearing in Pakistan is to provide sports (horse riding, polo, tent pegging, etc.) and draft power. They are often raised in proximity with ruminants at the small animal holders’ level, whereas separate at the well managed institutional level. Scarce literature is available regarding brucellosis in equines [18,19]. The purpose of this study was to update the existing knowledge and detect and differentiate the causative agent for brucellosis in breeding equines with reproductive disorders in Pakistan. 

## 2. Materials and Methods 

**Figure 1:**
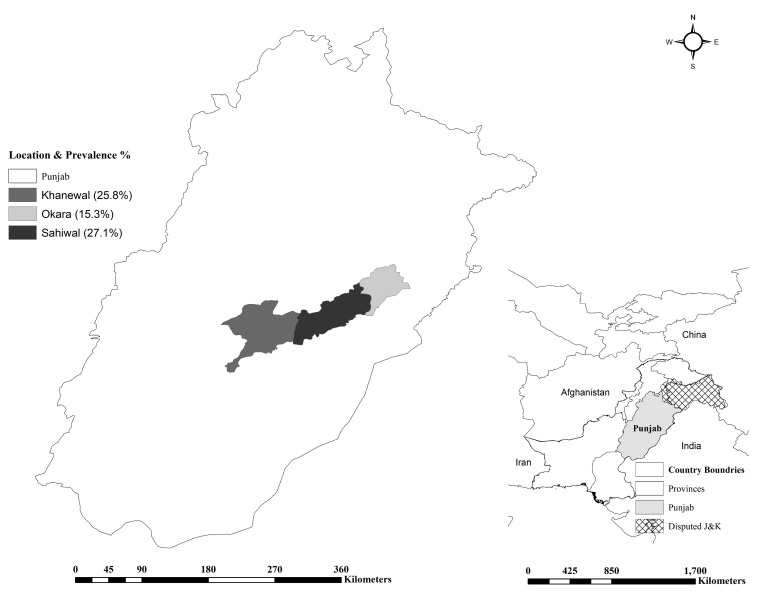
Map for equine brucellosis in Punjab, Punjab.

A total of 448 (427 mares, 13 stallions, and 8 Jackasses) of the 4564 “Ghori-Pal” equine population in the three prefectures of Punjab (Pakistan), including Okara (n = 977), Khanewal (n = 1204), and Sahiwal (N = 2383), were recruited. The sampling period spanned over 4 months from December 2017–March 2018 (Figure 1). These animals had a previous history of reproductive disorders, i.e., abortion (≤17% in pregnant mares) and infertility, but no history of articular bursitis (supra-atlantal bursitis, fistulous withers, hygromas, etc.). Owing to unavailability of prevalence estimates of equine brucellosis in the study locales, an expected prevalence of 50% with a 95% confidence interval (CI) and 5% desired absolute precision was taken for sample size estimation [20]. Thus, a minimum of 384 samples had to be taken from the equid population. Finally, 448 animals displaying clinical signs were sampled to be on the safe side. Blood samples (3 mL) were withdrawn directly into gel-clot serum vials. These equids had minimum to no contact with prevailing ruminant herds in the neighborhood. Animal related factors, e.g., age, sex, and breed and environment related factors, e.g., feeding, housing, and breeding practice were noted on a proforma invoice. 

The sera were tested with Pourquier^®^ Rose Bengale Ag (IDEXX, Montpellier, France) for Rose Bengal Plate Test (RBPT), followed by parallel testing with ID Screen^®^ Brucellosis Serum Indirect Multi-species (IDVet, Grabels, France) for indirect-Enzyme Linked Immunosorbent Assay (iELISA) and Complement Fixation Test (CFT) in the presence of positive and negative controls (provided by Friedrich-Loeffler-Institut (FLI), Jena, Germany) as per standard/manufacturer’s recommendations [7,21,22]. A titer >20 ICFTU/mL was considered as positive for CFT. These tests were applied to detect anti-smooth-lipopolysaccharide (LPS) antibodies produced by exposure to *B. abortus* and/or *B. melitensis*. 

DNA was extracted from serum samples by QIAamp DNA Mini Qiacube kit (Qiagen, Hilden, Germany) in the presence of *E. coli* controls. The DNA was detected/differentiated (*B. abortus* and *B. melitensis*) by using genus and species-specific primers in a real-time PCR system along with *B. abortus* (ATCC 23448) and *B. melitensis* (ATCC 23456) as positive controls [23]. Nuclease free water was used as a no template control (NTC). Briefly, decontamination for 2 min at 50 °C followed by initial denaturation for 10 min at 95 °C, 1 cycle each, denaturation for 25 secs at 95 °C, and 1 min at 57 °C for primers annealing and elongation, 50 cycles for each. A ≤38 cycle threshold (Ct) value was considered positive based on previous in-house validation experience [24]. 

Univariate and multivariate analysis was done to determine the association of the factors with the infection by using SPSS version 10.0 (IBM, Armonk, NY, USA) and a map was built using ArcGIS (Esri, Redlands, CA, USA).

Ethical statement: The animals were treated and handled for blood collection as per bio-ethical and standard procedures of the “Institutional Bio-Ethics Committee, University of Baluchistan, Quetta, Pakistan” (vide letter no. UOB/ORIC/19/258).

## 3. Results

Overall, 21.4% (96/448) samples were found positive by RBPT, 3.56% (16/448) by iELISA, and 4.24% (19/448) reacted in CFT (Table 1 and Table 2). The reproductive disorders’ history was highest in equines of Sahiwal followed by Khanewal and Okara (data unpublished). The equids were raised and maintained in a 1000 m^2^ stable area that comprised of a grazing and an open area paddock. The mares were first mated at four years of age by natural breeding. No artificial insemination was practiced. These equids were fed on gram (*Cicer arietinum*) and barley (*Hordeum vulgare*) grains, wheat bran and oats (*Avena sativa*), and lucerne (*Medicago sativa*) as green fodder. Based on previous published literature, we considered RBPT as the final criteria for statistical analysis. Univariable analysis indicated a higher occurrence in horses than in donkeys with a nonsignificant association (Table 1). Age was found to be significantly associated (*p* < 0.05), with the highest occurrence 9/22 (40.9%; CI 20.7–63.6) at early age (≤5 years), followed by 51/207 (24.6%; CI 18.9–31.1) at younger age (5.1–10 years) and 36/219 (16.4%; CI 11.8–22) at older age (>10 years). Animal sex showed a nonsignificant association (*p* > 0.05), however prevalence was numerically higher in females (22.3%; CI 18.4–26.5) than the males (4.8%; CI 0.1–23.8). Geographical location was found to be significantly associated (*p* < 0.05) with the occurrence of the infection, with the highest numbers being found in Sahiwal 48/177 (27.1%; CI 20.7–34.3), followed by 16/62 (25.8%; CI 15.5–38.5) in Khanewal and 32/209 (15.3%; CI 10.7–20.9) in Okara (Figure 1). Breeds showed a nonsignificant (*p* > 0.05) association. Local breeds showed the highest 5/10 (50%; CI 18.7–81.3) seroprevalence followed by exotic breeds 71/341 (20.8%; CI 16.6–25.5) and the Arabian breed, with 20/97 (20.6%; CI 13.1–30). Abortion was insignificantly associated (*p* > 0.05) with seroprevalence 1/3 (33.3%; CI 0.8–90.6) and 95/445 (21.4%; CI 17.6–25.5) in animals with a prior history. The PCR analysis of samples revealed the presence of *B. abortus* in all seropositive animals. 

In multivariate analysis, age and location were connected with higher risks, i.e., ≤5 years (OR 3.37; CI 1.32–8.60) followed by 5.1–10 years (OR 1.68; CI 1.04–2.73) and Sahiwal (OR 2.05; CI 1.03–4.09) followed by Khanewal (OR 1.99; CI 1.20–3.31) (Table 3).

Of the 448 samples analysed by three different serologic assays, 96, 16, and 19 samples tested positive, respectively, in RBPT, iELISA, and CFT (Table 2). Out of the 96 RBPT positive samples, nine were iELISA and one was CFT positive also. An inter-rater reliability analysis using the Kappa statistics was performed to determine agreement among three tests (Table 4). There was poor agreement between RBPT and iELISA results, k = 0.106 (95% CI 0.022, 0.19), *p* = 0.001. Whereas, there was no agreement between RBPT and CFT results, k = −0.057 (95% CI −0.114, −0.0005), *p* = 0.079. Poor agreement was also observed between iELISA and CFT results, k = 0.197 (95% CI 0.10, 0.29), *p* < 0.01.

## 4. Discussion

Since long ago, ruminant brucellosis has been endemic in Pakistan [25]. Although equines are not primary hosts of the disease, they are considered dead-end hosts [26]. Previously, *B. abortus* has been reported in equines elsewhere and as a cause of bovine brucellosis in the country [1]. Hence, we conducted this study to investigate the burden and etiology of equine brucellosis in Pakistani Punjab. 

Serology is the main stay in diagnosing brucellosis, which relies on a variety of tests, including RBPT, serum agglutination test (SAT), milk ring test (MRT), ELISAs, fluorescence polarization assay (FPA), and CFT. Each of the tests has certain limitation in terms of diagnostic accuracy. RBPT and iELISA are highly sensitive compared to CFT, which is widely considered as a specific test. Similarly, the SAT shows great promise in terms of sensitivity, with a quantitation ability, especially in humans. MRT is a sensitive and practical tool for both individual and bulk milk tank screening in bovines. Having poor sensitivity, PCR is a highly specific test, which is used as a complementary identification and typing tool. Real-time format is superior to conventional PCR, which provides better sensitivity while maintaining the specificity. Most of these tests require standardization and validation depending upon the disease situation in the area, laboratory conditions, and local resource power [27]. The bacterial culture is a gold standard for diagnosis of brucellosis; nonetheless, it is less sensitive, requires advanced biosafety conditions, e.g., biosafety level-III, has a long test turnover time, and possesses serious human health hazards. Therefore, we chose serology and PCR for the present investigation. 

A higher seroprevalence by RBPT may be ascribed to nonspecific reactions of the RBPT antigen to certain bacteria, e.g., *Salmonella, Yersninia*, and *E. coli* [28]. Since RBPT has been found to be a satisfactory diagnostic test in our previous reports from Pakistan, we considered RBPT results as final criteria and for statistical analysis [27,29]. 

This study did not identify statistically significant differences (*p* > 0.05) between horses and donkeys. However, a higher seroprevalence in horses (21.8%; CI 18–26) might be owing to the fact that the horses were overrepresented in the sample size. Moreover, comparatively more male horses were tested than male donkeys. This might have influenced the results and should be confirmed in further studies to evaluate the difference and influence of the species on results [18,30]. 

Age was found to be statistically significantly associated (*p* > 0.05) with a positive serological test (Table 1). Animals younger than <10 years were found to be more at risk (≤5 years, OR 3.37; CI 1.32–8.60 and 5.1–10 years, OR 1.68; CI 1.04–2.73) than older ones, i.e., >10 years (Table 3). These findings are supported by previous literature, suggesting the presence of antibodies without obvious signs at an earlier age and a decline in detectable antibody titer with growing age associated with latency [18,30]. 

Sex of the animals was not found to be significantly associated (*p* > 0.05) with positive serology. Females showed 22.3% (18.4–26.5) seroprevalence and males 4.8% (0.1–23.8). These findings may again be biased by the high number of female animals tested in this study because of the lower numbers of breeding horses in the population. A better comparison is possible when comparable numbers of both sexes can be recruited in studies. These findings are supported by previous studies, which found that abortive discharges and transmission of brucellosis were more often associated [11,12,18,30]. However, opposing arguments do exist.

Location was significantly associated (*p* < 0.05) with seroprevalence in this study (Table 1). The highest seroprevalence was noted in Sahiwal (27.1%; CI 20.7–34.3), followed by Khanewal (25.8%; CI 15.5–38.5) and Okara (15.3%; CI 10.7–20.9). The risk of occurrence was higher in Sahiwal (OR 2.05; CI 1.03–4.09) than the other two districts (Table 3). These results are in parallel with Wadood et al., who suggested management related factors to influence the results [18]. However, a significant association has also been found [31]. 

The breed was insignificantly associated (*p* > 0.05) with seropositivity, however a higher percentage of positives was recorded in local breeds (50%; CI 18.7–81.3) followed by exotic (20.8%; CI 16.6–25.5) and Arabian breeds (20.6%; CI 13.1–30). A similar pattern to that of Wadood et al. was found, with little variation [18]. However, a scientific explanation could not be found. 

Surprisingly, history of abortion was not significantly (*p* > 0.05) associated with the outcome of the infection (Table 1). Even a higher seroprevalence (33.3%; CI 0.8–90.6) was found in animals without prior history of abortion (21.4%; CI 17.6–25.5). Rarity of abortion is well known in equine brucellosis [11].

Our study has demonstrated the occurrence of *B. abortus* as the prevailing cause of brucellosis in the tested equines. Previously, *B. abortus* and to a lower extent, *B. suis* have been reported in equines [11]. Although equines were raised away from the ruminant herds, the infection might have transmitted via a route that could not be ascertained in this study. In Pakistan, endemicity of *B. abortus* is known in ruminants and the animals raised in close contact with ruminants have been reported to contract the infection from the existing herd [32]. Isolation of the etiologic agent(s) both from equines and ruminants could be helpful in determining if the same strain is causing the infection in this area. 

## 5. Conclusions

Livestock brucellosis remains an endemic problem in the country. Fistulous withers and poll evil cases should be treated with care as they could be hazardous and a source of transmission to humans. Lateral transmission of infection to other domestic animals is unknown. Eradication of brucellosis in ruminants will positively affect prevalence in dead-end hosts, such as horses. Age and location were found to be risk factors. Routine screening starting at an age of 12 months should be considered in the brucellosis control policy in equines. Males should be the focus of future studies for a better assessment of the epidemiological situation. *B. abortus* was found to be the cause of brucellosis in equines in Pakistan, but this finding needs further proof, probably by isolation of the bacteria.

## Figures and Tables

**Table 1:** Univariable analysis based on the RBPT results.

Variable	Category	Pos./Tested	Prev. % (95%CI)	*p*-Value	OR	95% CI
Species	Donkey	0/8	0 (0–36.9)	0.091 *	-	-
	Horse	96/440	21.8 (18–26)		-	-
Age	≤5 Y	9/22	40.9 (20.7–63.6)	χ^2^ = 9.464 *p* = 0.009	3.52	1.40–8.85
	5.1–10 Y	51/207	24.6 (18.9–31.1)		1.67	1.03–2.68
	>10 Y	36/219	16.4 (11.8–22)		Ref	-
Sex	Female	95/427	22.3 (18.4–26.5)	χ^2^ = 3.635 *p* = 0.057 *	5.72	0.76–43.19
	Male	1/21	4.8 (0.1–23.8)		Ref	
Location	Okara	32/209	15.3 (10.7–20.9)	χ^2^ = 8.755 *p* = 0.013	Ref	-
	Khanewal	16/62	25.8 (15.5–38.5)		1.92	0.97–3.81
	Sahiwal	48/177	27.1 (20.7–34.3)		2.06	1.25–3.39
Breed	ArabX	20/97	20.6 (13.1–30)	χ^2^ = 4.961 *p* = 0.084	Ref	-
	ExoticX	71/341	20.8 (16.6–25.5)		1.01	0.58–1.77
	Desi	5/10	50 (18.7–81.3)		3.85	1.02–14.61
Abortion	No	1/3	33.3 (0.8–90.6)	χ^2^ = 0.257 *p* = 0.614	1.84	0.17–20.53
	Yes	95/445	21.4 (17.6–25.5)		Ref	-
Total		96/448	21.4 (17.7–25.5)			

* calculated by Fisher’s exact test; Pos. = Positive; Prev. = prevalence; CI = Confidence Interval; OR = Odds ratio.

**Table 2:** Comparison of the results of RBPT, iELISA, and CFT used to detect anti-*Brucella* antibodies in equines (n = 448).

RBPT		iELISA		CFT	
		Negative	Positive	Negative	Positive
Negative	Observed	345	7	334	18
	Expected	339.4	12.6	337.1	14.9
Positive	Observed	87	9	95	1
	Expected	92.6	3.4	91.9	4.1
Total (Observed)		432	16	429	19

RBPT = Rose Bengal Plate Test; iELISA = indirect-Enzyme linked Immune sorbent Assay; CFT = Complement Fixation Test.

**Table 3:** Multivariable analysis based upon the RBPT results.

Variable	Exposure Variable	Comparison	OR	95%CI	*p*-Value
Age	≤5 Y	>10 Y	3.37	1.32–8.60	0.01
	5.1–10 Y	>10 Y	1.68	1.04–2.73	
Location	Sahiwal	Okara	2.05	1.03–4.09	0.02
	Khanewal	Okara	1.99	1.20–3.31	

Hosmer and Lemeshow Test Value = χ^2^ 3.216, 4 df, *p*-Value 0.522; Nagelkerke R^2^ = 0.059; OR = Odds ratio; CI = Confidence Interval.

**Table 4:** Agreement between RBPT, iELISA, and CFT used for serodiagnosis of brucellosis in equines (n = 448).

Comparison	Observed Agreement	SE	Kappa Value	95% CI of Kappa	*p*-Value	Strength
RBT vs. iELISA	79.02%	0.043	0.106	0.022, 0.19	0.001	Poor
RBT vs. CFT	74.78%	0.021	−0.057	−0.11449, −0.00049	0.079	No Agreement
iELISA vs. CFT	93.97%	0.097	0.197	0.100, 0.294	<0.01	Poor

RBPT = Rose Bengal Plate Test; iELISA = indirect-Enzyme linked Immune sorbent Assay; CFT = Complement Fixation Test; SE = Standard error.
